# A general approach for evaluating of the coverage, resolution, and representation of streamflow monitoring networks

**DOI:** 10.1007/s10661-023-11829-y

**Published:** 2023-09-30

**Authors:** Christopher P. Konrad, Scott W. Anderson

**Affiliations:** US Geological Survey, Washington Water Science Center, Tacoma, WA 98402 USA

**Keywords:** Streamflow monitoring, Network information, Monitoring design, Gap analysis

## Abstract

Streamflow monitoring networks provide information for a wide range of public interests in river and streams. A general approach to evaluate monitoring for different interests is developed to support network planning and design. The approach defines three theoretically distinct information metrics (coverage, resolution, and representation) based on the spatial distribution of a variable of interest. *Coverage* is the fraction of information that a network can provide about a variable when some areas are not monitored. *Resolution* is the information available from the network relative to the maximum information possible given the number of sites in the network. *Representation* is the information that a network provides about a benchmark distribution of a variable. Information is defined using Shannon entropy where the spatial discretization of a variable among spatial elements of a landscape or sites in a network indicates the uncertainty in the spatial distribution of the variable. This approach supports the design of networks for monitoring of variables with heterogeneous spatial distributions (“hot spots” and patches) that might otherwise be unmonitored because they occupy insignificant portions of the landscape. Areas where monitoring will maintain or improve the metrics serve as objective priorities for public interests in network design. The approach is demonstrated for the streamflow monitoring network operated by the United States Geological Survey during water year 2020 indicating gaps in the coverage of coastal rivers and the resolution of low flows.

## Introduction

Streamflow monitoring networks provide information to serve many different types of interests (WMO, 2008). Local interests in flood warning, planning and operation of water and wastewater infrastructure across many different sectors (transportation, energy, agricultural, industrial, residential), navigation, management of ecological resources, and recreation are often primary reasons for monitoring rivers and streams (Chang & Lin, 2014; Georgakakos, 1986; Jettmar et al., 1979; Konrad et al., 2012; Ning & Chang, 2003). While important, the need for streamflow information at particular locations has led to ad hoc development of streamflow monitoring networks (Wahl et al., 1995; Mlynowski et al., 2011; Normand, 2021), which may be neither cost-effective (Langbein, 1954; Strobl et al., 2006; Kristensen et al., 2012) nor support inference for unmonitored locations (Olsen et al., 1999; Wagner et al., 2008; DeWeber et al., 2014; Krabbenhoft et al., 2022).

Streamflow monitoring networks can be designed to better serve public interests (Parr et al., 2002; Squillace, 2020), but network design has often been approached as a multi-variate optimization that is particular to network objectives (Lanfear, 2005; Ning & Chang, 2003; Safavi et al., 2021). In reviewing analyses of monitoring networks, we identified coverage, resolution, and representation as three common design objectives, but they have not been used consistently to characterize gaps in monitoring networks (WMO, 2008; Wan et al., 2013; Thornton et al., 2022). Wagner et al. (2008) describe a method for assessing lake monitoring programs in terms of representation of the frequency of lakes over gradients in different types of land use. DeWeber et al. (2014) apply the method to assess the United States Geological Survey (USGS) streamflow monitoring network’s representation of various landscape characteristics. We generalize this method for any spatially conserved variable (counts, lengths, areas, volumes, or fluxes) and extend the scope of network analysis to the coverage and resolution of variables of interest.

**Fig. 1:**
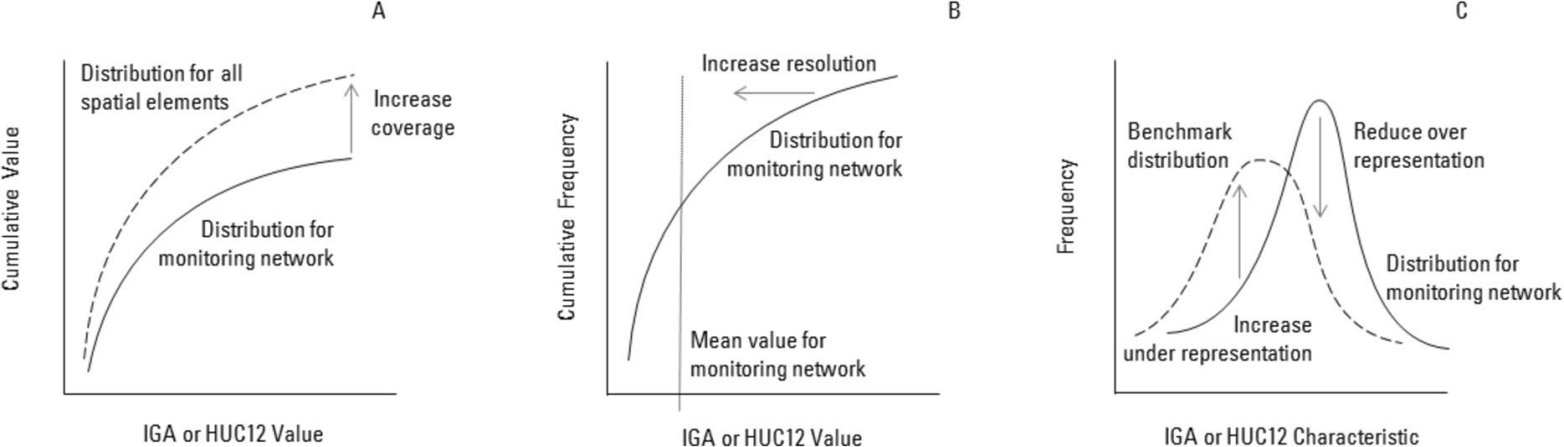
Network coverage indicated by the difference as the cumulative value of a spatially conserved variable for incremental gaged areas (IGAs) in network relative to the cumulative value for all spatial elements, which are 12-digit hydrologic unit code watersheds (HUC12s) for the application to the USGS network **A**, network resolution of a spatially conserved variable indicated by deviations of values for a monitoring network compared to the mean value for the network **B**, network representation indicated by the difference between the network and a benchmark distribution for a characteristic **C **with arrows showing changes in a monitoring network that will increase coverage, resolution, or representation

We propose theoretically distinct definitions of network coverage, resolution, and representation that lead directly to the identification of priority areas for monitoring (Fig. 1). *Coverage* is the maximum fraction of information that a monitoring network can provide about a variable because of unmonitored areas. Coverage neglects the spatial distribution of the variable in those unmonitored areas, which would increase the information about a variable in these areas and, thus, reduce the fraction of information provided by the network. *Resolution* is the fraction of information available from the monitoring network relative to the maximum possible information that can be acquired by a monitoring network with the same number of sites. *Representation* is the similarity of network information to a benchmark. With these definitions, coverage, resolution, and representation support hierarchical analysis of monitoring networks that comprehensively addresses different types of monitoring objectives.

Quantitative analysis of information requires a probability distribution to represent the uncertainty of a variable (Cover & Thomas, 2006; Shannon, 1948). In contrast to the information provided by hydrologic time series, which uses the frequency distribution of the values in the time series as probability (Amorocho & Espildora, 1973; Keum & Coulibaly, 2017; Sreeparvathy & Srinivas, 2020), our approach uses the fraction of a spatial variable associated with each spatial element (point, line segment, or polygon) in the landscape or with each site in a monitoring network as the general probability distribution (Renyi, 1961). In this framing, a monitoring network provides information about the source or location of a variable. Arguably, monitoring networks do not reduce uncertainty in the spatial distribution of any variable that is not monitored (e.g., stream length, land cover, administrative designations). Nonetheless, inferences involving unmonitored variables (e.g., the effects of climate on streamflow or management actions on water quality) are limited by the spatial discretization of these variables by the network.

## Methods

The objectives for the general approach are to calculate three metrics—network coverage, resolution, and representation—and to identify priority areas for maintaining or adding monitoring sites for a variable of interest. The variables can be streamflow observations or measurements acquired by a monitoring network (e.g., streamflow, loads of materials transported by a river), factors that influence streamflow quantity or quality (e.g., precipitation volume, forest area, reservoir storage), or administrative designations that affect land or water management (e.g., length of rivers impaired for water quality, area of land administered by native American tribes). Time series variables must be summarized by a statistic (i.e., mean annual flow, annual maximum temperature).

**Fig. 2:**
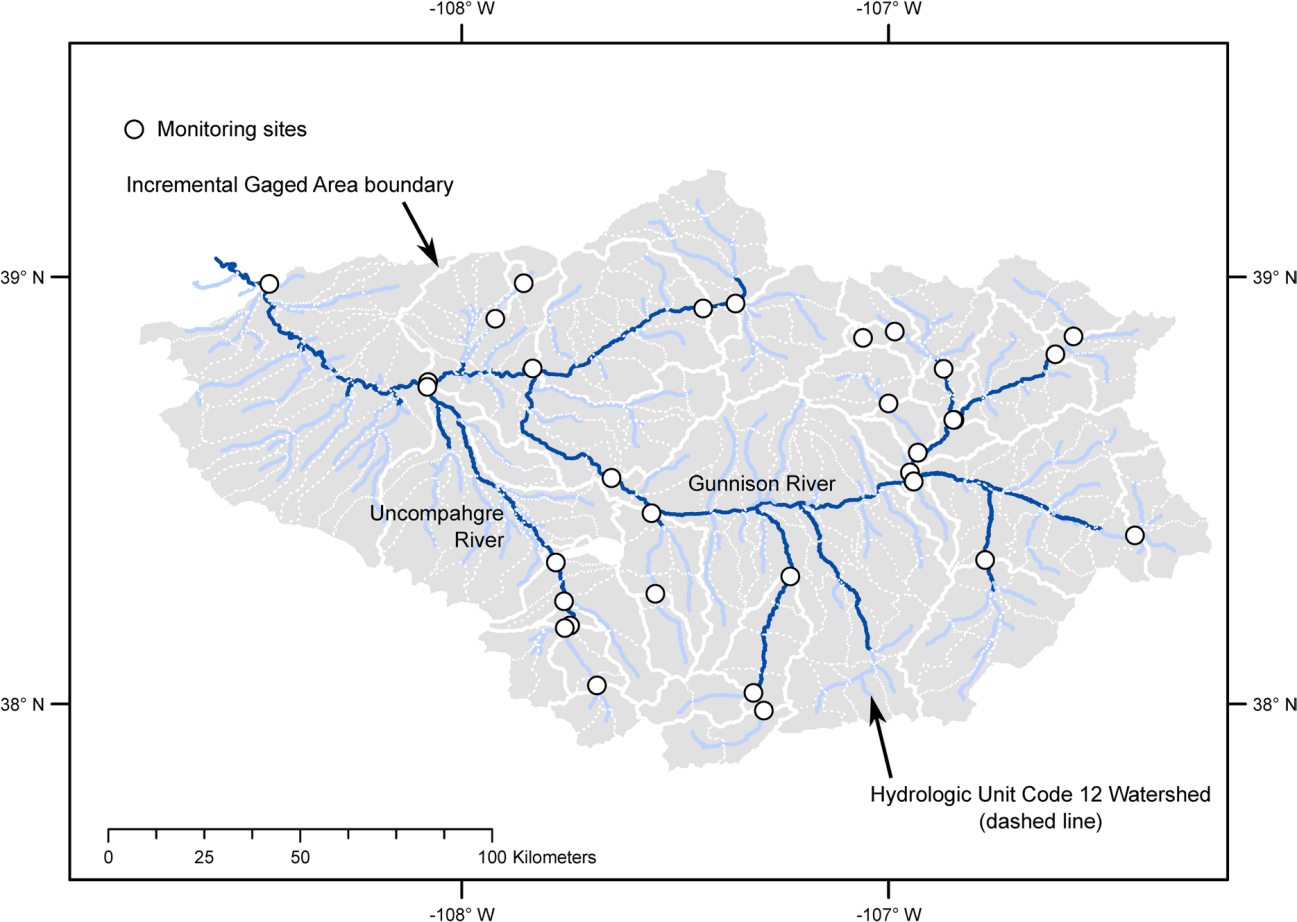
Spatial framework for the Gunnison River basin in Colorado. Rivers and streams with drainage areas greater than 500 km^2^ are shown with dark blue lines; streams with drainage areas between 50 and 500 km^2^ are shown with light blue lines

The approach requires a spatial framework (Fig. 2) that divides the domain of interest (an area or river system) into discrete, non-overlapping elements (points, line segments, or polygons). The elements may be determined by the location of sites in a monitoring network, the resolution of available data, or a pragmatic limit on monitoring density. The sites in the monitoring network must be assigned uniquely to an element with no more than one site per element. Spatial elements are assigned to the first downstream monitoring site and aggregated into an incremental gaged area (IGA) for each site. If there is no downstream monitoring site, the spatial element is designated as unmonitored. Variables are discretized spatially to create distributions of incremental values for the spatial element and IGAs.

An example of the spatial framework for the Gunnison River basin, Colorado is illustrated in Fig. 2. The Gunnison River drains a 20,500 km^2^ area on the west slope of the Rocky Mountains where snowmelt is the dominant source of streamflow; large reservoirs are used to store water as part of the water management system for the Colorado River, and irrigated agriculture is a dominant land use in the lower basin. The stream network in Fig. 2 has been simplified to only segments with drainage areas greater than 50 km^2^ for clarity. The basin comprises 231 12-digit hydrologic unit code (HUC12) watersheds that range in area from 33 to 171 km^2^ (USGS, 2022a). Variables derived from spatially continuous data sources (e.g., land cover, precipitation) are calculated for each HUC12 watershed to create cumulative distributions for the coverage metric (Fig. 1A) and benchmark distributions for the representation metric (Fig. 1C). The USGS streamflow monitoring network for the Gunnison River in WY20 had 35 sites that could be assigned uniquely to flowlines (stream segments) from the National Hydrography Dataset (NHD) Plus version 2 (Schwarz & Wieczorek, 2018). The IGAs associated with monitoring sites ranged in area from 5 to 3300 km^2^, so the IGA distributions used for resolution (Fig. 1B) and representation (Fig. 1C) can be expected to have wide variances for many variables.

### *Information-theoretic basis for network metrics*

Network metrics are defined as distinct measures of spatial information using entropy (Shannon, 1948). Entropy, H, quantifies the uncertainty of a variable, *V*, as,

1 $$\mathrm{H}\left(V\right)=-\sum\limits_{s=1}^{S}p\left({V}_{s}\right)\mathrm{ ln} p\left({V}_{s}\right)$$

where *V* = {*v*_1_, *v*_2_, *v*_3_, …, *v*_S_} is the set of values of *V* for spatial elements 1 to S. The variable *V* must be spatially conserved where its value aggregated over multiple elements is the sum of its value for individual elements (e.g., area of wetland but not wetland as a fraction of area). For a spatial conserved variable, the probability, *p* (*V*_*s*_), that *V* is located in or originates from element *s* is based on the fraction of *V* in element *s*,

2 $$p\left({V}_{s}\right)=\frac{|{v}_{s}|}{\sum |V|}$$

where *v*_s_ is the incremental value of *V* assigned to element *s* (not the value accumulated from multiple elements). If *V* is spatially conserved, Eq. (2) meets the requirements of Eq. (1) for a probability distribution that is a non-negative additive set function where Σ*p*(*v*_s_) = 1 (Renyi, 1961). The probabilities in Eq. (2) are calculated using absolute values to allow for fluxes that can be negative (e.g., losses of streamflow or net deposition of sediment along a river) and the interpretation of *p*(*v*_s_) must be expanded to the probability that *V* originates, is stored, or terminates in element *s* for these variables.

Entropy calculated from Eq. (1) using probability defined empirically in Eq. (2) represents the uncertainty in the distribution of *V* among spatial elements rather than the uncertainty in its value at a location. Empirical probability distributions maintain the generality of the approach and avoid the need for assumptions about a variable’s value at any location. This general approach accommodates conventional assessment of network representation of non-conserved variables (characteristics such as water temperature, land surface elevation, drainage area, fraction of area that is wetland), which rely on the cumulative distribution of a spatially conserved variable (site counts, stream lengths, land surface area).

The information that a streamflow monitoring network with *N* sites provides about a variable can be quantified as,

3 $$\mathrm{H}\left(W\right)=-\sum\limits_{n=1}^{N}p\left({W}_{n}\right) \mathrm{ln}\; p\left({W}_{n}\right)$$

where *W* = {*w*_1_, *w*_2_, *w*_3_, …,*w*_*N*_} is the set of values corresponding to sites 1 to *N* in the network, and the probability is defined as the monitored fraction of *W* assigned to site *n* using the values of a variable, *w*_*n*_, instead of *v*_*s*_ in Eq. (2), *p*(*W*_n_) =|*w*_n_|/Σ|*W*|. For a streamflow monitoring network, *w*_*n*_ = Σ *v*_s_ for all of the spatial elements *s* upstream of site *n* but downstream of other sites in the network (Clark et al., 1994; National Research Council 2004). We refer to the aggregation of spatial elements for each monitoring site as an incremental gaged area (IGA).

Theoretically distinct metrics of network coverage, resolution, and representation are developed from the entropies of a variable, H(*V*), and the network, H(*W*). The metrics have standardized ranges from 0 (no information) to 1 (maximum possible information) with linear scaling based on a variable’s value rather than log scaling of a variable’s probabilities used for entropy. The metrics form a hierarchy for evaluating network information but are not intended to be used indiscriminately as design objectives for every variable of interest.

### Network coverage

A monitoring network will have incomplete information about a variable, H(*W*) < H(*V*), where there are unmonitored spatial elements. The network coverage metric, *Cv*, is defined as the fraction of a variable that is monitored,

4 $$Cv = \frac{{\sum }_{n}|{w}_{n}|}{{\sum }_{s}|{v}_{s}|}$$

which indicates that the network is missing an information because of unmonitored spatial elements. The total value of a variable must be known or estimated from a model for areas where the values are unknown to calculate *Cv*. Coverage can vary from *Cv* = 0 (the variable is outside the spatial domain of the network) to *Cv* = 1 (the variable is completely within the spatial domain of the monitoring network).

A monitoring network is necessarily missing information when coverage, *Cv* < 1, because *p*(*Ws*) is an “incomplete” probability distribution (Renyi, 1961) that is missing values for unmonitored spatial elements. The upper limit on the information provided by a network with incomplete coverage can be calculated from *Cv* for the case when only one spatial element, *S*, is outside the monitoring network, and otherwise, the network provides complete information about *V* such that

5 $$\mathrm{min}\left[\mathrm{H}\left(V\right)-\mathrm{H}\left(W\right)\right]=p\left({v}_{S}\right)\mathrm{ ln }\;p\left({v}_{S}\right)$$

where *p*(*v*_*S*_) = 1 − Σ*W*/Σ*V* and, by substituting *p*(*v*_*S*_) = 1 − *Cv*,

6 $$\mathrm{min}\left[\mathrm{H}\left(V\right) -\mathrm{H}\left(W\right)\right]=\left(1-Cv\right)\mathrm{ ln}\left(1-Cv\right)$$

Although *Cv* indicates the upper limit on information that a network can provide about a variable (Eq. 6), it does not account for the spatial distribution of a variable within the network or the landscape, which are addressed, respectively by network resolution and representation.

### Network resolution

The spatial resolution of a network for a variable is the smallest value that can be distinguished (National Institute of Standards & Technology, 2012), which typically varies over the spatial domain of the network; resolution of the variable, *W*, is relatively high for IGAs where values are less than the mean value per site (*w*_*n*_ < Σ*W*/*N*) but is relatively low for IGAs where values are greater than the mean value per site (*w*_*n*_ > Σ*W*/*N*). A monitoring network with a fixed number of sites, *N*, maximizes spatial information about the sources of a spatially conserved variable when the variable is discretized by the network into equal increments. In this case, the value for each IGA is the mean value of the variable per IGA, and the probability that *W* originates from any spatial element is *p*(*w*_*n*_) = 1/*N* (Cover & Thomas, 2006). For example, a monitoring network maximizes information about the sources of streamflow when the network divides an area into IGAs that contribute equal amounts of streamflow.

The network resolution metric, *Rs*, is defined here as the deviation of the absolute values of *W* from the mean absolute value weighted by the fraction of the total absolute value of the variable,

7 $$Rs=1-\frac{\sqrt{\sum_{n}|\left|{w}_{n}\right|-\stackrel{-}{\left|{w}_{n}\right|}|\left|{w}_{n}\right| }}{\sum |W|}$$

to indicate the information available from a particular network of *N* sites relative to the maximum information network with *N* sites. The absolute values in the formulation of *Rs* accommodate variables that have negative values. Network resolution ranges from *Rs* ~ 0 when a variable is only observed at one site to *Rs* ~ 1 when all sites have about equal values.

Information about a variable outside of the spatial domain of the monitoring network is not incorporated in *Rs*, so *Rs* complements *Cv* without redundancy. *Rs* is a relative measure of resolution; it does not indicate the increase in network resolution from adding sites. The median (or maximum) value of *W* is an alternative to indicate the absolute resolution of a network. While discretization of a spatially conserved variable into equal values will maximize the information acquired by a monitoring network about its spatial distribution, it does not assure that the network will represent variation in other characteristics of interest.

### Network representation

Information from a monitoring network can be used to make inferences about landscapes or river systems, if network sites collectively represent characteristics of the landscape (e.g., land surface elevation and land cover) or characteristics of rivers (e.g., water temperature, channel gradient, or streamflow). Network representation of a characteristic typically is evaluated by comparing the frequency distribution of the characteristic for the network to a benchmark distribution. In both cases, frequency is the fraction of a spatially conserved variable (i.e., counts, stream lengths, area) with a given value (or range) of the characteristic (DeWeber et al., 2014; Kiang et al., 2013; Laize, 2004). The difference between the cumulative distributions for the benchmark and the network indicates the network’s “efficiency” (Cover & Thomas, 2006) in providing information about the spatial distribution of the characteristic.

The spatial elements of the benchmark and the network may not have matching values of the characteristic, so the difference between frequencies cannot be calculated directly (e.g., Kullback & Leibler, 1951). Instead, the difference between network and benchmark distributions is approximated by the difference of histograms with the same class intervals created from the distributions. The benchmark distribution is divided by its deciles into 10 intervals, each interval containing 0.1 Σ*V*, the spatially conserved variable for the benchmark. The values of the characteristic, *C*, for the deciles of the benchmark distribution define the class intervals, {min(*C*), *c*_1_, *c*_2_, …, *c*_*d*_…, max(*C*)} where *c*_*d*_ is the value for the *d*th decile, of the histogram for the network. If the network represents the benchmark perfectly, then each class interval will have 0.1 Σ*W*, the spatially conserved variable for the network. A negative (positive) deviation of the frequency of a class interval for the network from 0.1 indicates under (over) representation of that interval. Network representation is then defined as,

8 $$Rp=1-\frac{1}{1.8}\sum\limits_{d=1}^{10}\left|\frac{{\sum }_{n}{w}_{n}}{\sum W}-0.1\right|$$

where n is the set of elements of W with characteristic values c_d-1_ < c_n_ < c_d_. The factor 1/1.8 standardizes representation from *Rp* = 0, when the ranges in the characteristic for the benchmark and the distribution do not overlap to *Rp* = 1 when each bin of the network histogram has a frequency of 0.1.

### Application to the USGS streamflow monitoring network

The workflow for the analysis is implemented as a series of scripts in the statistical programming language R (Konrad et al., 2022). The workflow was applied for the national USGS streamflow monitoring network and repeated for each major river basin in the USA (USGS, 2022b). An initial set of 9362 sites where USGS collected daily streamflow for at least 182 days during water year 2020 were identified in the National Water Information System (USGS, 2021). Gages on canals, lakes, or estuaries were excluded. To delineate incremental gaged areas, monitoring sites in the contiguous United States (CONUS) were assigned to flowlines (stream segments) in NHD (Schwarz & Wieczorek, 2018), which identifies the downstream flow line for each flow line in CONUS. Monitoring sites in outside of CONUS were assigned to HUC12 watersheds and use the routing information (downstream HUC12) from the Watershed Boundary Dataset (Konrad et al., 2022; USGS, 2022a). Multiple sites are located on a single NHD flow line in CONUS (or any HUC12 in non-CONUS areas); only the furthest downstream site was retained for analysis. The final network for the analysis has 8113 sites: 7034 sites are nested in the drainage area of a downstream gage, and 1079 gages are terminal with no downstream site.

### Spatial framework for the USGS streamflow monitoring network

Incremental gaged areas (IGAs) were delineated by aggregating the catchments of NHD flowlines upstream of each monitoring site and downstream of any other site for CONUS and aggregating HUC12s for each site in non-CONUS areas. Because of the limited spatial resolution of NHD catchments and HUC12 watersheds, IGA boundaries are only approximation of watershed boundaries and generally are shifted downstream from the sites defining an IGA (USGS, 2022b). Routing of NHD flowlines includes divergent flow at distributary nodes, which was simplified for this analysis by using only the primary flowline at any distributary node. Primary flowline designations were changed downstream of some distributary nodes to better represent the drainage area of sites. HUC12 routing does not include distributaries, so it did not have to be simplified, but it was edited to correct loops and gaps in routing.

### Spatial variables and characteristics

Geospatial data sets representing a wide range of public interests in streamflow monitoring including streamflow and material loads transported by rivers, physical and anthropogenic influences on streamflow and water quality, and administrative designations of lands and waters were compiled from publicly available source (Konrad et al., 2022; National Oceanic & Atmospheric Administration, 2020; National Wild & Scenic Rivers System, 2021; US Environmental Protection Agency, 2014; USGS, 2000, 2014a, b; Wieczorek et al., 2018). In many cases, the results are limited to CONUS due to the coverage of the source dataset. Sixteen individual variables or characteristics (Table 1) and three regional classification systems with multiple categories (Table 2) were selected for analysis to address common interests in streamflow monitoring. Spatially conserved variables were summed by IGA or HUC12. Characteristics were averaged over area or stream length of IGAs and HUC12s. A spatially conserved variable was identified for the frequency distribution of each characteristic (Table 1). Each category of the classification systems (357 climate divisions, 85 ecoregions, and 50 types of surficial geology) was analyzed individually using the area of the category as the spatially conserved value and the fraction of IGAs or HUC12s as the factor for representation. Rather than presenting the results for every category of these classification systems, the results are consolidated for each system using a single “coverage-representation” criterion to identify categories as having gaps in network coverage or representations when less than 25% of the area of the category is comprised by IGAs that are predominately (at least 90%) in the category.

**Table 1:** Coverage, resolution, and representation of 16 variable or characteristics of interests the US Geological Survey streamflow monitoring network

Variable/characteristic	Spatially conserved variable	Coverage (Cv)	Resolution (Rs)	Representation (Rp)
Drainage area^a^	Area	0.75	0.95	0.54
Incremental median annual minimum daily streamflow^a^	Streamflow	NA	0.79	NA
Incremental median annual maximum daily streamflow^a^	Streamflow	NA	0.95	NA
Coastal streams^a^,*	Length	0.17	0.90	0.75
Minimum monthly temperature < 0 °C^b^,*	Area	0.89	0.96	1.0
Urban land cover^b^,*	Area	0.75	0.96	0.77
Wetlands^b^,*	Area	0.74	0.95	0.85
Streams listed as impaired for water quality^f^	Length	0.87	0.97	0.61
Major sources of wastewater discharges^b^,*	Count	0.78	0.96	0.88
Wild and Scenic Rivers^g^	Length	0.72	0.91	0.57
Federal Wilderness^d^	Area	0.21	0.82	0.38
Land administered by Bureau of Land Management^c^	Area	0.58	0.85	0.51
Land administered by US Forest Service^c^	Area	0.83	0.96	0.65
Land administered by native American tribes^e^*	Area	0.92	0.88	0.46
Population^b^,*	Area	0.74	0.97	0.81
Reservoir storage as years of mean streamflow^h^,*	Area	0.84	0.97	0.74

*Variable or characteristic is limited to CONUS

^a^Konrad et al. (2022)

^b^Wieczorek et al. (2018)

^c^USGS (2014a)

^d^USGS (2000)

^e^USGS (2014b)

^f^US Environmental Protection Agency (2014)

^g^National Wild and Scenic Rivers System (2021)

^h^US Army Corps of Engineers (2013)

**Table 2:** Summary classification systems for climate divisions, ecoregions, and surficial geology categories with gaps in coverage or representation

System	Number of categories	Number of categories where less than 25% of area is covered by IGAs that are at least 90% in the category
Climate divisions^a^	357	96
Ecoregions^b^,*	85	16
Surficial geology^c^,*	50	46

*Variable or characteristic is limited to CONUS

^a^National Oceanic and Atmospheric Administration (2020)

^b^Wieczorek et al. (2018)

^c^Wieczorek et al. (2018)

Median annual values of minimum and maximum daily streamflow for water years 1981–2020 were calculated at sites with at least 5 years of daily streamflow (Konrad et al., 2022). Most active gages (7309/8113) have at least 5 years of daily streamflow record, but median annual statistics at sites with less than the full 40-year period are likely to have greater influence from hydroclimatic variability. Incremental values of median annual minimum streamflow (IncrQmin) and median annual maximum streamflow (IncrQmax) were calculated by differencing the values at upstream gages from the value at the downstream gage. The incremental values may not be reliable where upstream and downstream gages do not have overlapping periods of record. IGAs where the downstream gage had less than 5 years of record were merged downstream. Large negative values were inspected and generally were associated with reservoirs or withdrawals. Streamflow statistics were not estimated for HUC12s and, as a result, coverage and representation metrics (Fig. 1A and C) could not be calculated.

## Results and discussion

**Fig. 3:**
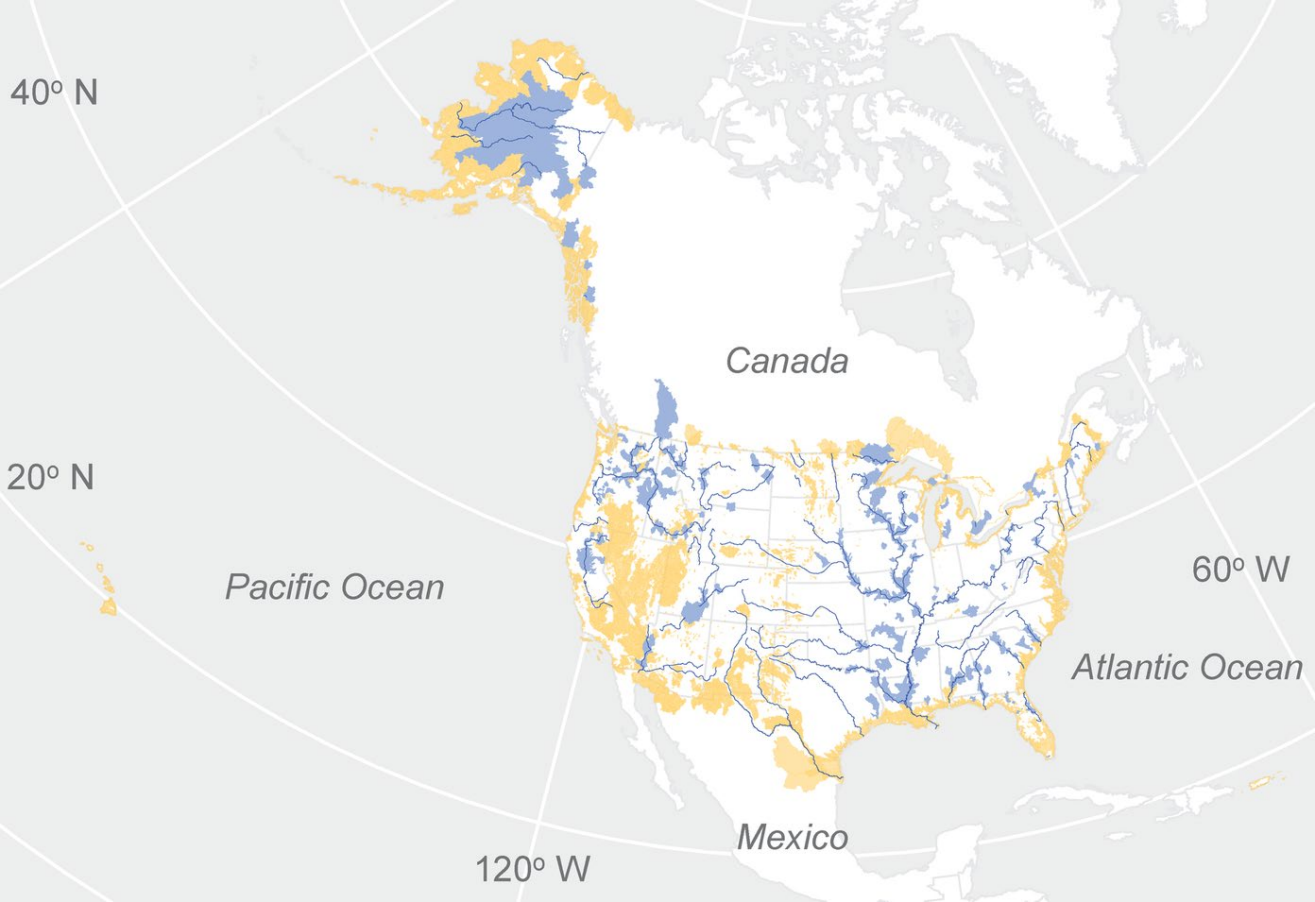
Incremental gaged areas in the USA and bordering areas where the absolute value of incremental median annual minimum daily streamflow > 12.7 m^3^/s (blue areas) indicating low network resolution, 12-digit hydrologic unit code watersheds where more than 50% of their area is unmonitored (orange areas) indicating gaps in network coverage, and major rivers with drainage areas > 10,000 km.^2^ (blue lines)

**Fig. 4:**
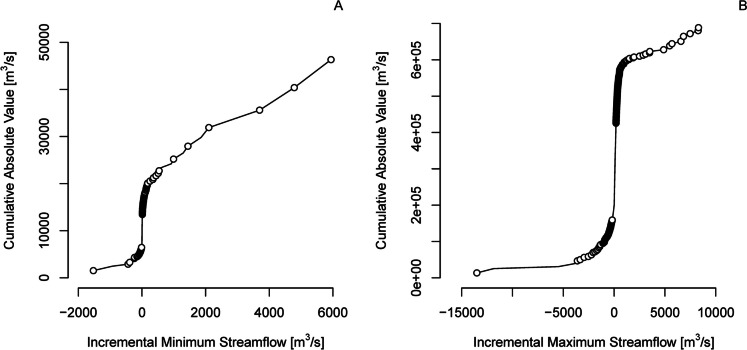
Cumulative distributions of incremental median annual minimum daily streamflow **A **and incremental median annual maximum daily streamflow for incremental gaged areas (IGAs) of the USA **B**. Priority IGAs for monitoring are shown with circles

**Fig. 5:**
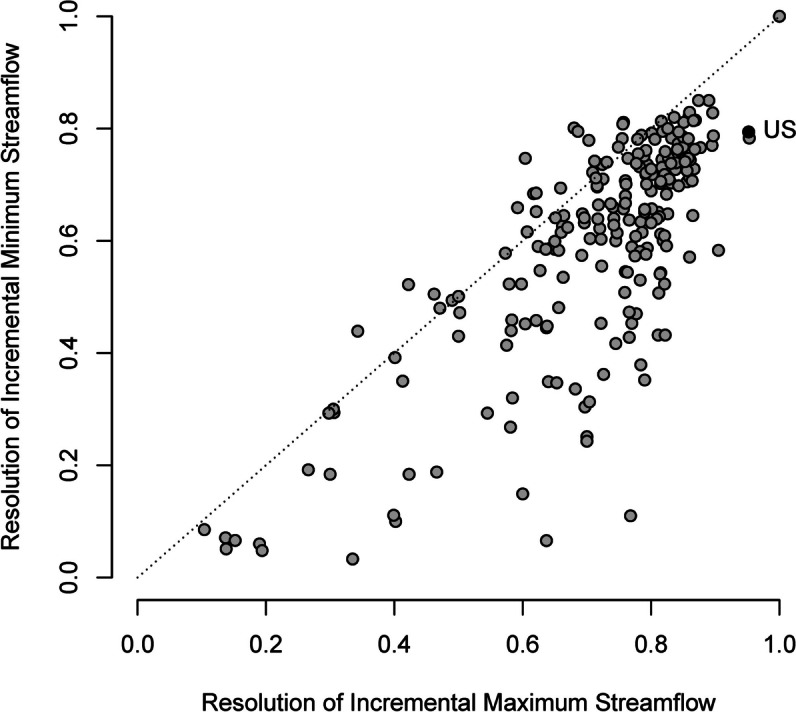
Comparison of the resolution of incremental median annual maximum and minimum daily streamflow and daily streamflow for the USGS streamflow monitoring network in 220 major river basins and the USA as a whole

The USGS streamflow monitoring network covers 7.4 million km^2^ of the USA (*Cv* = 0.75 for area) providing comprehensive streamflow information for the nation, supporting the development of continental-scale hydrologic models, and serving as a foundation for flood and drought warning systems. Geographic gaps in network coverage include portions of Alaska, the interior West, and coastal watersheds (Fig. 3, USGS, 1998; Kiang et al., 2013). The USGS network’s spatial resolution of streamflow is worst for low flows (*Rs* = 0.79 for incremental median annual minimum daily streamflow, Fig. 4A) and best for high flows (*Rs* = 0.95 for incremental median annual maximum daily streamflow (Fig. 4B). The lower resolution of the network for low flows is indicated by the large deviations of sites in the network from the mean value of 6.4 m^3^/s for incremental median annual minimum daily streamflow. In contrast, incremental median annual maximum daily streamflow is close to the mean value of 94 m^3^/s for most sites in the network (Fig. 4B). The difference reflects flood information as a primary network objective but also the positively skewed spatial distribution of the sources of low flow; the IGAs for St. Clair, Niagara, and St. Lawrence Rivers, which drain the Great Lakes, account for 30% of the incremental low flows for the USA. Likewise, the resolution of low flows is less than the resolution of high flows for 85% (188/220) major river basins (Fig. 5) because of spatial concentration in the dominant sources of low flow including groundwater discharge to rivers, snow and ice melt, lakes, and reservoirs (Konrad, 2006).

**Fig. 6:**
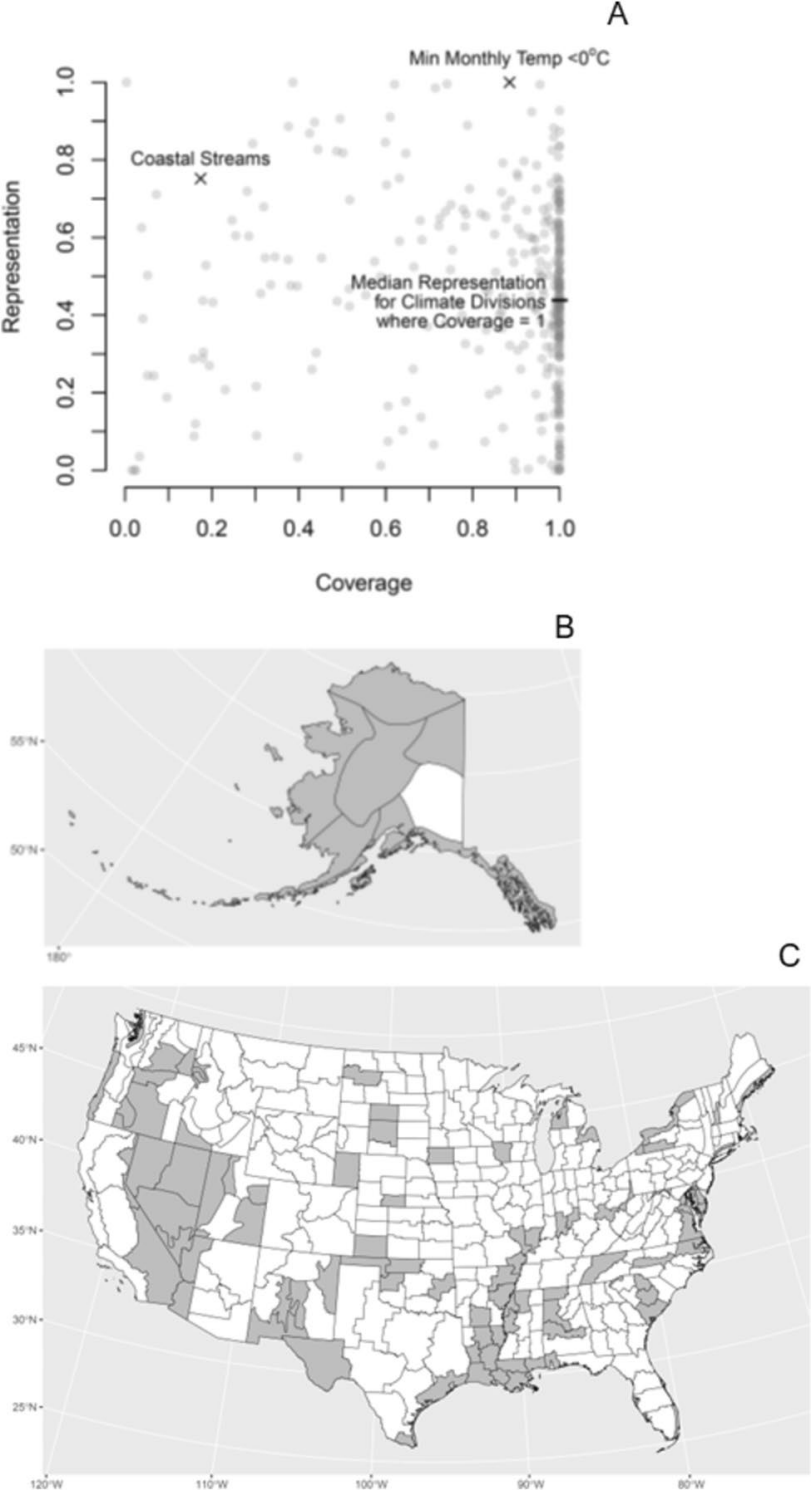
Coverage and representation of NOAA Climate Divisions for CONUS and Alaska (gray points), coastal streams for CONUS, and areas where minimum monthly temperature is less than 0 °C (min monthly temp < 0 °C) in CONUS **A **and climate divisions with “coverage-representation” gaps (gray) where less than 25% of the division area is comprised by incremental gaged areas predominantly (> 90% of their area) in that division for Alaska **B **and CONUS **C**

Major geographic gaps (Alaska, coastal watersheds, and interior West, Fig. 3) and a lack of gages on smaller rivers and streams limit the USGS network’s capability to monitor hydrologic responses to climate change across the USA. Network coverage is less than 50% (*Cv* < 0.5) for 51 NOAA climate divisions (Fig. 5A) and is very low e (*Cv* = 0.17) for coastal areas in CONUS (Fig. 6A; Table 1). Many climate divisions with low coverage do not necessarily have low representation (Fig. 6A) as long as their IGAs are located primarily in one division. Conversely, climate divisions where all rivers and streams are gaged (*Cv* = 1) can still have low representation (median *Rp* = 0.43). As a result, 108 climate divisions are identified as monitoring gaps because they do not meet the “coverage-representation” criterion where less than 25% of the division is comprised by IGAs that have more than 90% of their area in the division (Fig. 6B and C, Table 2). These climate divisions either have low coverage because their area is unmonitored or low representation because the IGAs that cover them include other climate divisions.

The USGS network has relatively high coverage (*Cv* = 0.89) and representation (*Rp* = 1) for areas where minimum monthly temperature is less than 0 °C (Fig. 6A), though there may be local gaps particularly where higher elevation snow and ice melt are critical for water availability. In this case, network representation of the elevation distribution for the USA or even a major river basin would not indicate whether the network provides information about high elevation catchments that occupy a small fraction of the landscape. Instead, “high elevation area” or “snowpack” would need to be analyzed as new variables to assess network information.

For areas in CONUS where minimum monthly mean temperature is less than 0 °C, the network has relatively high coverage (*Cv* = 0.89) and resolution (*Rs* = 0.96) (Fig. 5A) and essentially perfect representation (*Rp* = 1) (Table 1) because minimum monthly mean temperature is uniformly less or greater than 0 °C within IGAs and HUC12s; 99% of the gaged area where minimum monthly temperature is less than 0 °C is in IGAs where all of the IGA has minimum monthly temperatures less than 0 °C, and 91% of all area is in HUC12s where all of the HUC12 has a minimum monthly temperature less than 0 °C.

**Fig. 7:**
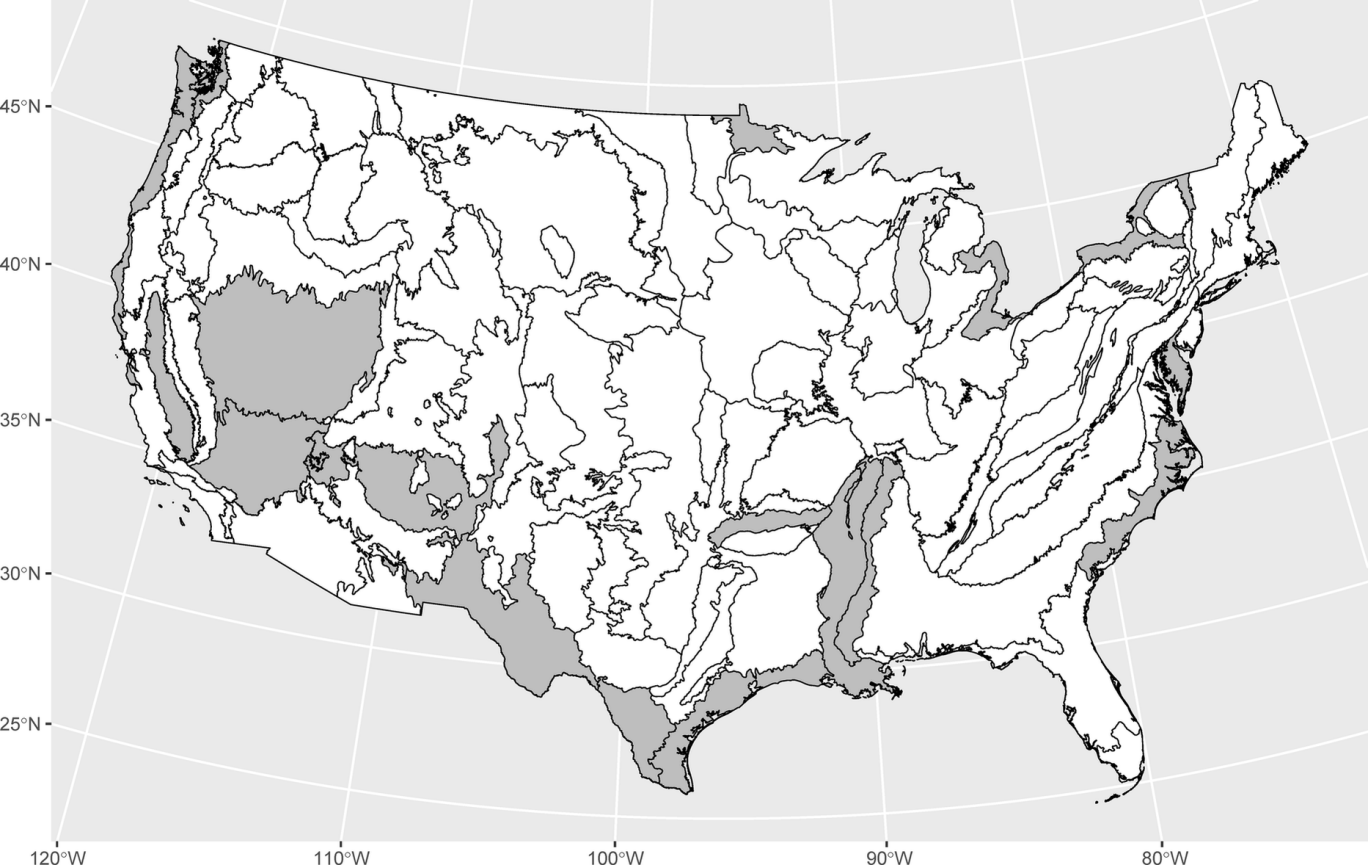
Level 3 ecoregions for CONUS with “coverage-representation” gaps (gray) where less than 25% of the ecoregion area is comprised by incremental gaged areas predominately (> 90% of their area) in that ecoregion

Most (70/85) level III ecoregions in CONUS meet the coverage-representation criterion. The 15 ecoregions that do not meet the coverage-representation criterion (Table 2) are in the interior West and along coasts, international borders, and the lower Mississippi River (Fig. 7). Only one type of surficial geology meets the coverage-representation criterion largely because lithologic contacts are not often aligned with watersheds, so most IGAs have mixtures of surficial geologies (Table 2).

Network representation of threats to the integrity of aquatic ecosystems in CONUS varies from *Rp* = 0.61 for rivers and streams listed under the Clean Water Act as impaired for water quality to *Rp* = 0.88 for major wastewater discharges permitted under the National Pollutant Discharge Elimination System (Table 1). The network generally has low representation of locations likely to have high ecological integrity including federally designated Wilderness Areas (*Rp* = 0.38) and National Wild and Scenic Rivers (*Rp* = 0.57) (Table 1). Likewise, the network does not fully represent reservoir regulation (*Rp* = 0.74, Table 1), both because of a lack of gages on both unregulated rivers and highly regulated rivers (e.g., reservoir storage > 0.5 mean annual streamflow).

Streamflow information acquired by the USGS network is used by other federal agencies and native American tribes to manage water resources. Network resolution of lands administered by United States Forest Service, Bureau of Land Management (BLM) and Tribes is greater than 0.8, but representation is less than 0.7 (Table 1, see Konrad et al., 2022 for other federal agencies). Low representation is a result of mixed administration of IGAs compared to HUC12s, which often are dominated by a single entity.

Network resolution and representation are greater than 0.7 for all types of land cover except snow/ice in CONUS (Konrad et al., 2022). Land cover types with lower values of representation indicates spatial fragmentation of that type at a scale smaller than most IGAs but not necessarily smaller than most HUC12s. For example, IGAs with urban development (*Rp* = 0.77, Table 1) generally have mixed land cover and, thus, only moderate fractions of urban development even where there are large urban areas. Some HUC12s have higher fractions of urban development, so network representation is relatively low for urban development. In contrast, network representation of wetlands is higher (*Rp* = 0.85, Table 1) because wetlands generally are much smaller than HUC12 watersheds, so few HUC12s have a high fraction of wetland cover. In both cases of urban development and wetlands, benchmark distributions based on smaller spatial elements than HUC12s would lower network representation.

### Network representation of variables with heterogeneous spatial distributions

**Fig. 8:**
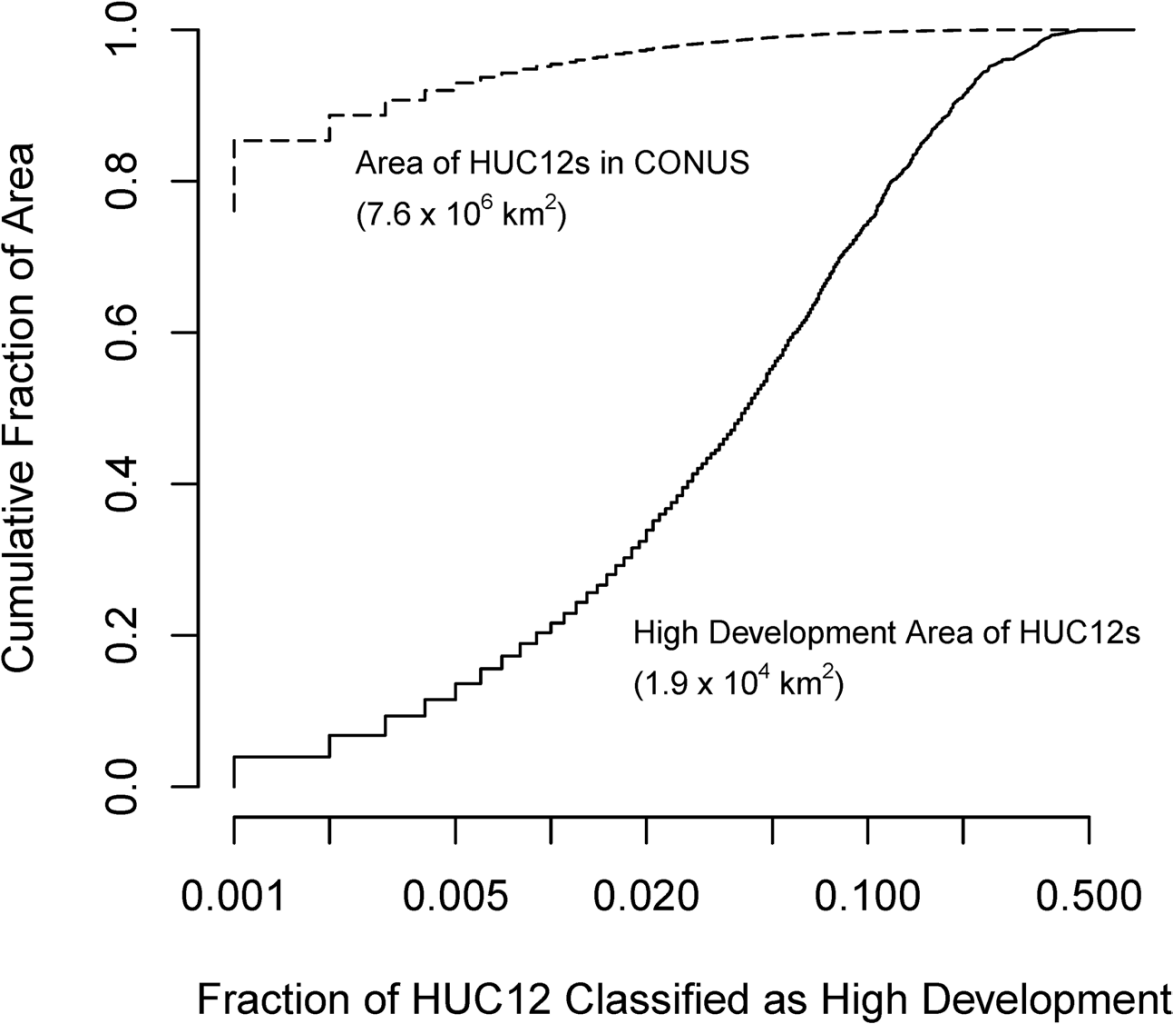
Spatial concentration of high development (urban) area for the contiguous United States (CONUS) in 12-digit hydrologic unit code watersheds (solid line) compared to the fraction of CONUS area with high development (< 25%, dashed line)

Conventional spatial frequencies, defined as the fraction of points, lengths, or areas with a characteristic value (Batty, 1974; Kiang et al., 2013; Poff et al., 2006; Wagner et al., 2008), indicate the relatively low likelihood of encountering features that are concentrated in “patches” or “hot-spots” such as wetlands or urban development. As a result, these features may not be incorporated into monitoring networks designed to represent the landscape. In contrast, the probabilities calculated from Eq. 2 explicitly account for the fraction of a variable in spatial elements, and thus, its spatial concentration. Conversely, spatial elements with no or low values of a variable provide little information about the variable according to Eq. 2. Thus, information about a spatial variable depends on the probability of where a variable is located and not the probability of its value at a location. These contrasting distributions lead to profoundly different benchmarks for network representation for variables that concentrated in space such as urban development (Fig. 8). Less than 0.1% of CONUS is classified by the National Landcover Data Set as “high development” (Dewitz, 2019), which is used here for urban land cover. Half of the high development area in CONUS is found in HUC12s with at least 4.2% high development (Fig. 8, solid line), but these HUCs comprise only 1% of the area of CONUS (Fig. 8, dashed line). Thus, a network representing land cover for CONUS would have few sites with any high development while a network representing how high development occurs in the landscape as cities would need sites that nominally have at least 4% high development in their watersheds.

A pragmatic benchmark distribution for network representation of a variable must balance the spatial discretization necessary to depict homogeneous areas with high or low values (“patches”) by the size of elements that would be feasible to monitor. For the example of urban development, a cumulative distribution based on 30 m resolution of land cover data is an impractical standard for monitoring streamflow. For the USA, HUC12 watersheds, which have a median area of 90 km^2^, are sufficient to depict stream basins with hydrologically significant fractions of urban development (> 10%) and represent spatial units that conceivably could be monitored (Booth & Konrad, 2017; Konrad & Booth, 2005).

### Evaluation of recent network changes in the Delaware River basin

**Fig. 9:**
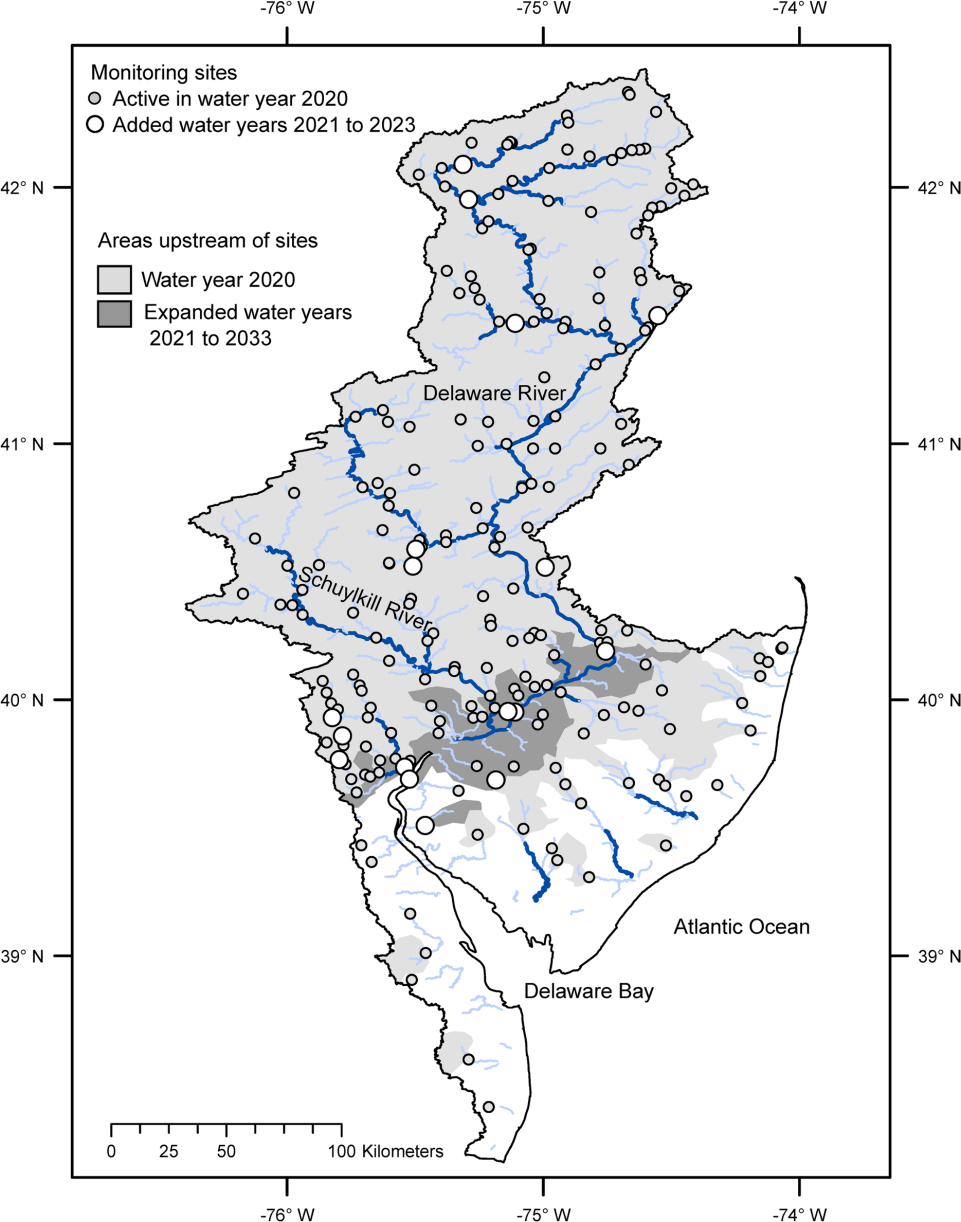
Map of the Delaware River basin showing gaged areas in water year 2020 and 2023. Rivers and streams with drainage areas greater than 500 km^2^ shown with dark blue lines, streams with drainage areas between 50 and 500 km^2^ shown with light blue lines

The Delaware River basin has been a focus for investment in monitoring through the USGS Next Generation Water Observing System (NGWOS) program (Murdoch et al., 2022). The workflow for network analysis was re-applied to the streamflow monitoring network in the Delaware River basin that was active during in water year 2023, which has 19 additional sites with continuous monitoring of streamflow compared to the previous 203 sites active in water year 2020 (Fig. 9, site counts based on a maximum of one gage per NHD flowline, gages on diversions are not included). In this case, network metrics provide objective measures of how monitoring investments have improved streamflow information even though the metrics were not used as design objectives. The additional sites expand network coverage of areas that drain to the lower main stem of the Delaware River, which increases the coverage metrics by at least 0.1 for coastal streams, low and high development, withdrawals, population, major NPDES discharges, three types of surficial geology, three ecoregions, and two climate divisions (Table 3). Network resolution increased by at least 0.1 for coastal streams. Network representation increased by at least 0.1 for coastal streams. Although network expansion increased the coverage of many variables, it reduced resolution or representation of some of these (e.g., high development areas). The lower values of resolution and representation, however, do not indicate network degradation because the number of gages increased. Instead, they are relative measures indicating greater potential resolution or representation in the expanded network.

**Table 3:** Comparison of selected metrics for the streamflow monitoring network in the Delaware River basin in water year 2020 (WY20) and water year 2023 (WY23)

Variable	Coverage		Resolution		Representation	
	WY20	WY23	WY20	WY23	WY20	WY23
Coastal streams	0.13	**0.28**	0.76	0.67	0.42	**0.62**
Low and medium development	0.61	**0.80**	0.89	0.84	0.80	0.80
High development	0.50	**0.86**	0.87	0.73	0.71	0.60
Withdrawals	0.72	**0.90**	0.87	0.83	0.77	0.77
Population	0.62	**0.87**	0.88	0.81	0.75	0.76
Major NPDES	0.58	**0.84**	0.82	0.76	0.85	0.86
Alluvial sediments less than 100 feet thick	0.23	**0.53**	0.71	0.62	0.56	0.51
Residual materials developed in igneous and metamorphic rocks	0.84	**1.00**	0.72	0.74	0.72	0.64
Residual materials developed in fine-grained sedimentary rocks	0.55	**0.69**	0.84	0.83	0.80	0.81
Middle Atlantic Coastal Plain Ecoregion	0.11	**0.26**	0.53	0.60	0.52	0.34
Northern Piedmont Ecoregion	0.79	**1.00**	0.19	**0.33**	0.56	0.63
Atlantic Coastal Pine Barrens Ecoregion	0.42	**0.56**	0.79	0.79	0.53	0.50
Northern Delaware Climate Division	0.23	**0.47**	0.66	0.58	0.30	0.35
Northern New Jersey Climate Division	0.40	**0.56**	0.79	0.78	0.81	0.70

Values in bold changed by at least 0.1 from WY20 to WY23

### Coverage, resolution, and representation as distinct types of network information

Spatial gaps in monitoring networks have been described variously as deficiencies in network coverage (e.g., Ning & Chang, 2003; Thornton et al., 2022), density (e.g., Coulibaly et al., 2013), or representation (e.g., Laize, 2004, DeWeber et al., 2014). Indeed, even the objective of maximizing temporal streamflow information acquired from a monitoring network (Alfonso et al., 2010; Caselton & Husain, 1980; Foroozand & Weijs, 2021) can be viewed as maximizing network coverage of entropy of a temporal variable. In this case, the incremental value for a site is the conditional entropy of its streamflow over time given the joint entropy of streamflow across all other sites in the network.

Coverage, resolution, and representation can be defined as theoretically distinct metrics (Eqs. 4, 7, and 8) such that any one metric does not depend on the others as demonstrated for coverage and representation of climate divisions in the USA (Fig. 6A). The distinction between network coverage and resolution is not common in network analysis beyond surface water: a monitoring network of widely spaced groundwater wells or meteorological stations does not necessarily “cover” intervening areas (Wan et al., 2013; Thornton et al., 2022). In contrast, surface water observations at a site generally are considered to provide information about its entire watershed such that the watershed is “covered” by monitoring at its outlet even as the information may not be representative of smaller catchments in the watershed or any particular type of watershed (Kiang et al., 2013; Laize, 2004). Monitoring network density (WMO, 2008) incorporates network coverage and suggests network resolution but does not differentiate among different spatial configurations the same number of monitoring sites over a given area of interest. Density is particularly limited as a useful metric for streamflow monitoring networks because of spatial autocorrelation of streamflow, loads, and watershed conditions. Distances between sites can be used as an alternative to indicate spatial resolution of a monitoring network but require an underlying model for spatial autocorrelation (Ning & Chang, 2003).

Streamflow monitoring generally is biased toward larger, perennial rivers and under-represent smaller streams (DeWeber et al., 2014; Krabbenhoft et al., 2022; Poff et al., 2006). The “large-river” bias is evident in the USGS network’s representation of HUC12 drainage area (*Rp* = 0.54, Table 1), but it extends to rivers and streams with drainage areas as large as 800 km^2^. Monitoring smaller streams can improve network representation of spatially fragmented features such as urban areas, wetlands, or habitats of endangered species, advance understanding of non-perennial flow dynamics, and support modeling. A monitoring network, however, may not be able simultaneously to represent different features with distinct spatial distributions. In this case, a more feasible objective for network design would be a network that has sites where features of interest are concentrated in various combinations (Kristensen et al., 2012; Murdoch et al., 2022) as a starting point for experimental designs that use subsampling (Chaloner & Verdinelli, 1995; Munn et al., 2018; Vaisman, 2020) or models that accommodate multi-collinearity of features across sites (Graham, 2003; Tasker & Stedinger, 1989).

### Priority areas for monitoring

Coverage, resolution, and representation are not intended as objectives for every variable of interest in network design; these are different types of network information that may require different designs to address as demonstrated for the Delaware River basin (Table 3). Where one of these metrics is an objective for a variable, priority monitoring areas can be identified objectively based on how the metric will respond to network changes. Adding monitoring sites in unmonitored areas (where there are no downstream sites) will increase network coverage if the variable of interest is present in those areas. Adding a monitoring site to an IGA where a variable’s value is greater than 2 × the mean value of the variable per site can increase network resolution by creating two IGAs with values closer to the mean. Priorities for network resolution are an empirical analog to “critical sampling points” determined from spatially distributed models of loading to streamflow (Strobl et al., 2006). Adding a site with a characteristic value in an unrepresented portion of the benchmark distribution will increase network representation of that characteristics. Likewise, criteria for maintaining sites follow from the changes in metrics that would result if monitoring is discontinued at a site (USGS, 2022b).

**Fig. 10:**
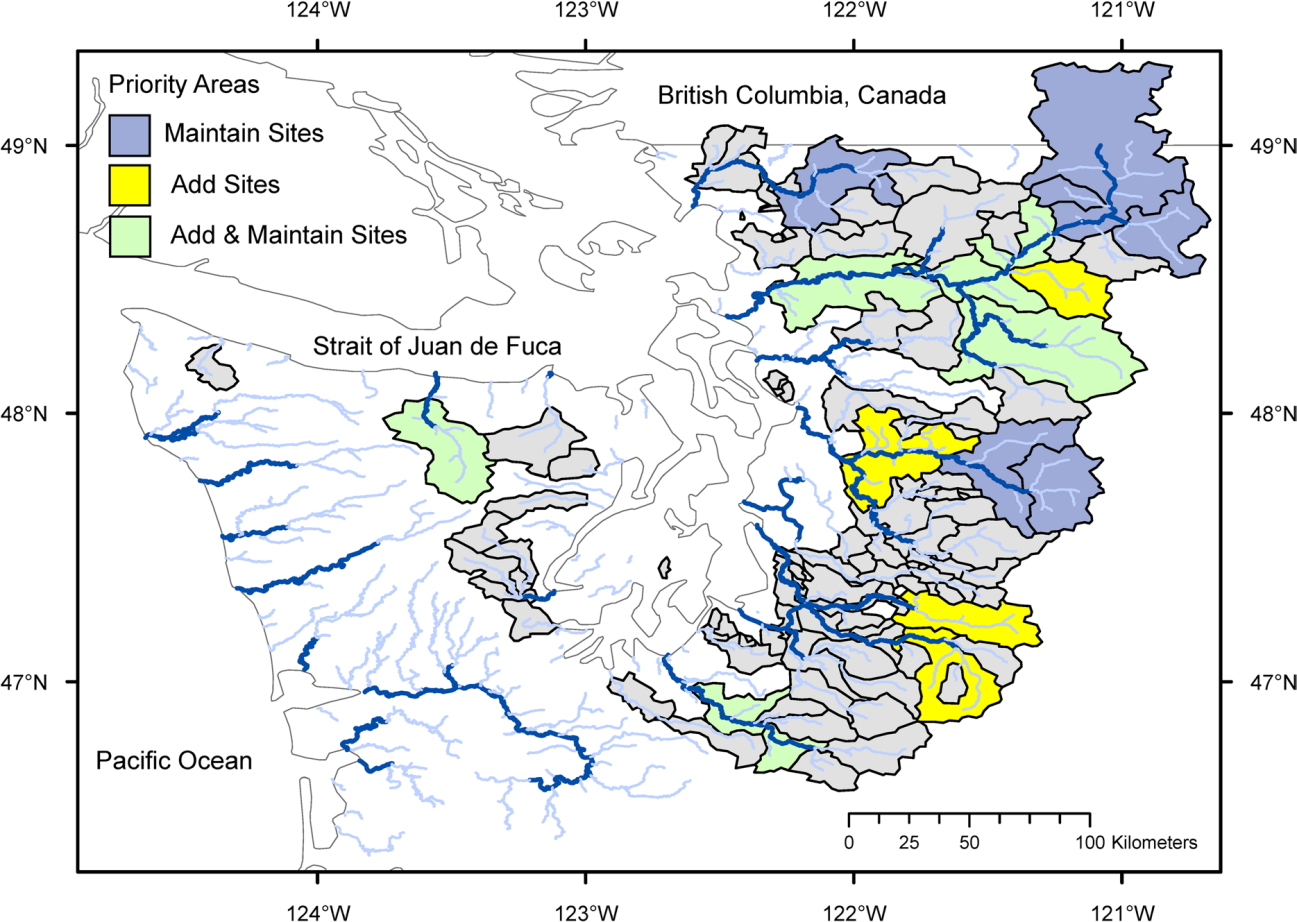
Priority monitoring areas for spatial resolution of low flows in the Puget Sound basin. Rivers and streams with drainage areas greater than 500 km^2^ are shown with dark blue lines; streams with drainage areas between 50 and 500 km^2^ are shown with light blue lines. Gray areas are gaged but their incremental median annual minimum streamflow IncrQmin < 6.2 m.^3^/s (the criterion for the “add” priority)

Priority areas to maintain or add monitoring sites can be easily explained as having high values of a variable or values that are under-represented by a current monitoring network. For example, monitoring sites in IGAs in the Puget Sound basin, Washington are priorities to maintain for low flows if incremental median annual minimum streamflow IncrQmin > 8 m^3^/s and are priorities to add monitoring sites if IncrQmin > 6.2 m^3^/s (Fig. 10). In this case, the priority of any IGAs meeting the “add” criterion was changed to “maintain” if they have recently established sites where IncrQmin cannot yet be estimated. IGAs not identified as priority areas are still important for monitoring, but there may be alternative sites that would provide equivalent network coverage, resolution, or representation.

The evaluation and design of monitoring networks with multiple types of objectives present a challenge because of trade-offs among objectives when selecting monitoring locations (Laize, 2004; Ning & Chang, 2003; Taheri et al., 2020). Heuristic valuation of explanatory and response factors is frequently used for network design (Burn & Golter, 1991; Chang & Lin, 2014; Lanfear, 2005; Strobl et al., 2006). Alternatively, a network can be designed to maximize the information acquired by monitoring (Caselton & Husain, 1980; Foroozand & Weijs, 2021; Krstanovic & Singh, 1992; Mishra & Coulibaly, 2010) or minimize the sample error of a model calibrated with that information (Fiering, 1965; Kiang et al., 2013; Marcus et al., 2003; Moss & Karlinger, 1974; Tasker & Stedinger, 1989). In all of these cases, network evaluation is conditioned on a weighting system, a single hydrologic variable, or a particular model. There is no assurance that the optimal network designed from these methods serves any interest well or all interests adequately, and the qualifications of the optimal network may need to be decomposed to understand how interests are served by the design (Barcellos & Souza, 2022; Fahle et al., 2015; Parr et al., 2002).

The univariate approach to network design developed here can be used to identify different configurations of monitoring sites in a network that provide equivalent information for a specific public interest. Network planning for multiple objectives would still require a way to balance or optimize over different interests (e.g., representation of drainage areas and resolution of streamflow). The combination of priority areas for multiple interests can quickly lead to an outcome where every area is a priority; in which case, network objectives may need to be specified more narrowly. More importantly, the alignment of local needs with priority monitoring areas for various public interests represents opportunities for garnering wide support for monitoring.

## Conclusions

Streamflow monitoring networks provide information for diverse public interests in rivers and streams. The capability of a network to serve these interests depends on its coverage, resolution, and representation of the spatial distribution of different types of monitored and unmonitored variables. We developed a general approach for network analysis that is scalable from river basins to continents. The approach is generalized for any spatial variable by using the observed distribution of the variable among spatial elements in landscape or network. It accommodates monitoring of “hot-spots” or “patchy” variables that might otherwise be unmonitored because they occupy insignificant portions of the landscape. Three theoretically distinct types of network information (coverage, resolution, and representation) are needed to identify gaps and priority areas for common objectives in the design of monitoring networks. Monitoring limited to priority areas will not maintain network coverage, resolution, and representation, but there may be alternative network configurations outside of priority areas that will provide equivalent information for public interests.

Application of the approach to the streamflow monitoring network operated by USGS demonstrates the challenges of addressing many different interests but provides transparency about where monitoring serves multiple interests and can be used to identify where local needs for streamflow information are aligned with broader public interests. In general, coverage of the USGS network would be improved by adding sites to unmonitored rivers and large streams in coastal areas, Alaska, and the interior West but more than 13,800 independent rivers and streams in the USA terminate at an ocean, estuary, or in closed basins. Comprehensive and feasible strategies to providing information in these unmonitored areas require hydrologic modeling in concert with monitoring.

Given the primacy of hazards and water availability for the USGS mission, gaps in network coverage in coastal areas and in network resolution of low flows are notable. Gaps in coastal areas are particularly significant because of increasing flood hazard from sea-level rise and exposure of growing populations to flooding. Although gaps in network coverage for Alaska and the interior West may not affect many people directly, they do limit understanding of hydrologic responses where climate is changing rapidly (Alaska) and where growing aridity may have severe social and ecological impacts (interior West). Otherwise, the network has capability to provide information about hydrologic responses to climate change in most climate divisions and colder areas in CONUS where an increasing fraction of precipitation is likely to be rain instead of snow. Network resolution of low flows, which is relatively poor compared to high flows, could be improved by synoptic low-flow surveys that target sources of baseflow such as lake outflow, groundwater discharge, and meltwater from snowfields, rather than adding continuous streamflow gages.

## Data Availability

The code used for the analysis, source data, and complete results are available (Konrad et al., 2022).

## Declarations

Use of product names do not imply endorsement by the US Government.
